# Maternal Tuberculosis Leading to Congenital Tuberculosis: A Case Report

**DOI:** 10.7759/cureus.39178

**Published:** 2023-05-18

**Authors:** Manidipa Barman, Binita Poudel, Ajmal Salam, Hari Prasad

**Affiliations:** 1 Pediatrics, All India Institute of Medical Sciences Rishikesh, Rishikesh, IND; 2 Emergency Medicine, All India Institute of Medical Sciences Rishikesh, Rishikesh, IND

**Keywords:** tuberculosis, mycobacterium tuberculosis, maternal, congenital, antitubercular drugs

## Abstract

In India, tuberculosis (TB) is a severe public health concern. We report a case of a 45-day male baby who had respiratory distress and fever, whose mother was diagnosed with pulmonary TB infection prior to delivery which was confirmed by a positive Cartridge-Based Nucleic Amplification Test (CBNAAT) from the sputum and was on antitubercular therapy (ATT). Due to the symptoms, signs, and maternal TB history, congenital TB was strongly suspected. A positive CBNAAT result from the gastric lavage further supported this suspicion. This case emphasizes the value of obtaining details on the mother's TB history to aid in the early diagnosis of congenital TB and expedite the treatment and prognosis.

## Introduction

Although tuberculosis (TB) is a common disease that affects people of all ages, only 468 cases of congenital TB have been reported globally as of 2009, with an infant mortality rate as high as 40% [1,2]. The reasons for the rarity of this disease in newborns are likely owing to the absence of clear-cut separation between congenital and acquired cases and late clinical suspicion if an ill-appearing infant does not improve with broad-spectrum antibiotics [3]. TB is a leading source of illness and death in India. More than 1400 people die from TB every day, killing around 480,000 people annually in India [4]. Congenital TB is the result of contracting Mycobacterium TB either during pregnancy or at delivery. Clinical features of congenital TB may be present at birth. However, they generally appear during the second or third week of life. The most frequent symptoms and signs are respiratory distress, fever, hepatic or splenic enlargement, poor feeding, lethargy, irritability, abdominal distension, failure to grow, ear discharge, and skin abnormalities [5].

## Case presentation

At 39 weeks of pregnancy, a 45-day-old male was born to an Indian mother via spontaneous vaginal delivery, weighing 2,500 grams. Delivery was uneventful, and the baby cried soon after birth. The baby did not require any admission to the nursery or neonatal intensive care unit and was discharged home with the mother. The baby was breastfed exclusively since birth, roomed in with the mother, and appeared symptom-free until the end of the fourth week. He presented to our hospital with complaints of low-grade fever for one week with no rash. He also had a dry cough for one week with no history of posttussive vomiting, difficulty breathing in the form of fast breathing for three days, and poor weight gain since birth. His mother, a 27-year-old primigravida, who was on regular antenatal visits, gave a history of cough from three months prior to delivery and was diagnosed subsequently as a case of pulmonary TB at 26 weeks of gestation, which was confirmed by a positive Cartridge-Based Nucleic Amplification Test (CBNAAT) from the sputum and was on antitubercular therapy (ATT) as per National Tuberculosis Elimination Program (NTEP) in India.

On admission, the baby was ill-appearing and noted as tachycardic (heart rate of 180 beats per min) and tachypneic (respiratory rate of 71 breaths per min) with baseline oxygen saturation of 88% on room air with subcostal and intercostal retractions. On auscultation, there was decreased air entry and coarse crepitation on the right side of the chest.

**Figure 1:**
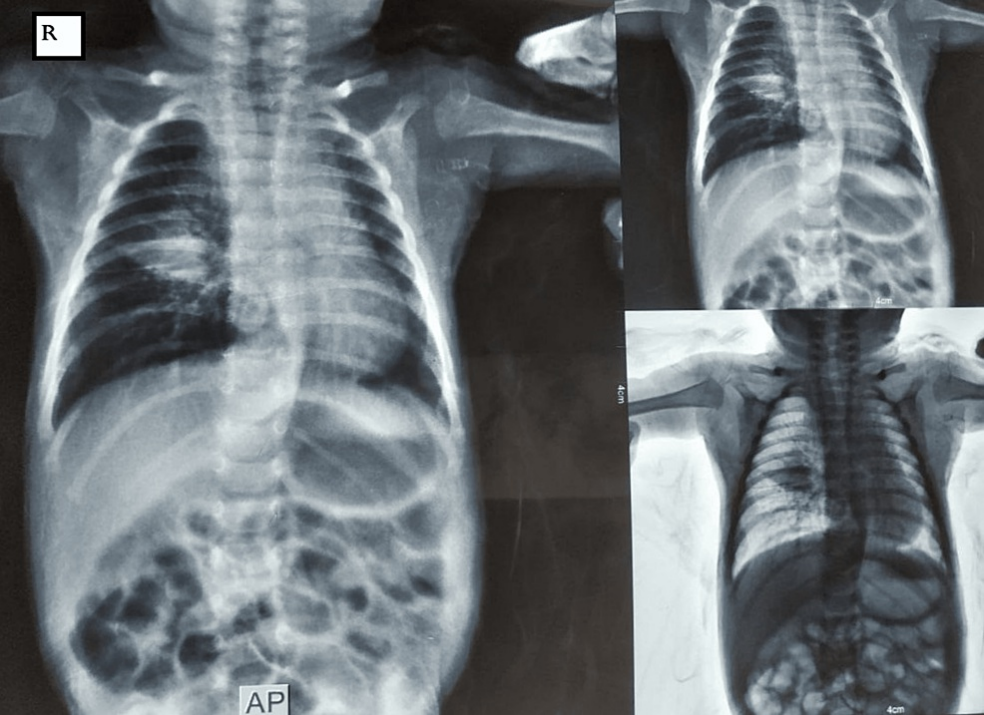
Chest x-ray showing infiltrates on the right upper and lower lobes

The blood investigations showed neutrophil-predominant leukocytosis (a cell count of 15,020/mm^3^ with 71.48% neutrophils). Chest x-ray on admission showed right-sided upper and lower lobe infiltrates, as in Figure 1.

He was empirically started on intravenous antibiotics linezolid (at 20 mg/kg/day) and piperacillin-tazobactam (at 300 mg/kg/day) and was continued for 21 days suspecting sepsis. The cerebrospinal fluid (CSF) examination was done in view of sepsis, and it revealed 120 cells (90% polymorphs and 10% monomorphs). CSF sugar and protein were 57 mg/dL and 177 mg/dL, respectively. CSF adenosine deaminase (ADA) was 4.9, and CSF CBNAAT did not detect TB bacilli. All the above findings were suggestive of tubercular etiology. But the gastric aspirate sent for CBNAAT was positive for rifampicin-sensitive TB bacilli; hence, the baby was diagnosed as a case of congenital TB with pulmonary involvement and was started on ATT as per Indian guidelines. After initiating antitubercular treatment, the infant's symptoms and signs improved gradually, and hence was discharged on ATT on day 22 of admission. The infant is now clinically well and is maintaining regular follow-ups in the outpatient department and is adherent to therapy- daily fixed drug combination (FDC) of ATT containing isoniazid, rifampicin, pyrazinamide, and ethambutol for two months. It is followed by four months of isoniazid, rifampicin, and ethambutol.

## Discussion

Congenital TB is a rare but serious illness that must be diagnosed immediately to prevent adverse outcomes. There are not many case reports of this illness in the literature. The placenta or the mother's vaginal tract may become infected during pregnancy due to tuberculous bacilli. Although vertical transmission is rare, the prognosis is not excellent. The infection may then spread to the developing infant by vaginal or placental infection [6].

Congenital TB can be transferred transplacentally if the major complex is in the liver. If the primary complex is in the lungs or digestive tract, congenital TB can be transmitted by aspirating infected amniotic fluid or other materials [7]. The most often affected organs are the lungs, liver, and spleen, which can result in fever, coughing, breathing issues, hepatomegaly, jaundice, splenomegaly, and abdominal distension. The brain, ear, lymph node, and skin are examples of other organs [8].

Up until 1989, there were only 300 cases documented in the literature; 58 patients were then examined in 1994 by Abughali et al. [9], and from 2001 to December 2005, 18 other cases were reported [2]. The most recent report of congenital TB to be recorded was covered in a review by Yeh et al. [1] between 2011 and 2019. In this, there were 16 cases of congenital TB (80%), the majority of which were recorded in Asian countries with a female predominance (male to female ratio of 9:11). The main clinical findings included respiratory distress in 71% of patients, fever in 67%, hepatosplenomegaly in 38% of cases, and cough in 33% of cases. Poor feeding, lethargy, lymphadenopathy, and seizures are some non-specific symptoms. Chest radiographs suggested pneumonia in 76% of the cases, numerous pulmonary nodules in 43%, and a milliary pattern in 38%. Only three patients presented with spleen and liver lesions, and two had pleural effusion [5]. According to a review, the death rate ranged between 33.9% and 52.6% between 1994 and 2009.

Beitzke created diagnostic criteria for distinguishing congenital TB from postnatal TB in 1935, and Cantwell refined them in 1994 [6]. The updated criteria demand infants with confirmed TB lesions and at least one of the following conditions:

(1) lesion that occurs in the first week of life,

(2) a cascading granuloma or main complex in the liver,

(3) TB infection of the placenta or maternal vaginal tract, and

(4) a careful examination of contacts to rule out postnatal transmission.

According to NTEP India, management begins with a daily FDC of ATT containing isoniazid, rifampicin, pyrazinamide, and ethambutol for two months. It is followed by four months of isoniazid, rifampicin, and ethambutol [10].

Any infant who exhibits bacterial or congenital infection-related signs and symptoms and whose response to supportive and antibiotic therapy is poor should be suspected of having congenital TB. Maternal or familial history of TB is the primary clue for an early diagnosis of congenital TB. Congenital TB continues to have a significant death rate due to delayed diagnosis. Many children may recover entirely if the diagnosis is recognized early and appropriate therapy is initiated.

## Conclusions

This case reiterates the importance of eliciting a contact history of TB, particularly maternal TB in an infant. Clinical diagnosis of TB is frequently challenging in pregnant patients and newborns due to the non-specific clinical presentation, necessitating a high index of suspicion. We urge a comprehensive investigation of TB evidence in mothers of newborns suspected of having congenital TB. Early detection followed by early initiation of ATT will improve outcomes.
